# Diseases of the Maxillary Bones and Their Periosteum

**Published:** 1894-04

**Authors:** Vida A. Latham

**Affiliations:** F.R.M.S.; Chicago, Ill.


### THE DENTAL REGISTER.

VOL. XLVIII.] APRIL, 1894. [No. 4.

### COMMUNICATIONS.

### Diseases of the Maxillary Bones and their Periosteum.

Read in the Section on Dental and Oral Surgery, at the Forty-fourth Annual Meeting of the American Medical Association.

BY VIDA A. LATHAM, F.R.M.S., D.D.S., CHICAGO, ILL.

Under this heading we will notice some points of interest connected with dental surgery and pathology, although the cause and history of these diseases are so numerous that it will be almost impossible to do more than briefly allude to some of the most common forms met with by dental surgeons, every one of whom ought to have such a knowledge of the affections of the oral cavity that they might, with some degree of certainty, determine what the causes are, their pathology, diagnosis, and their treatment. Especially should we know the morbid growths which can occur, whether benign or malignant, so that we may early diagnose the case, and if we do not care to take such cases for treatment, to warn our patient to consult a surgeon.

Unhappily, the question as to whether dentistry is a specialty of medicine still exists. Even such a man as Prof. Norman W. Kingsley says in the *Medical Record*, Nov. 20, 1886: "Oral surgery, even in its most comprehensive sense, is not dentistry, any more than dentistry in its most comprehensive sense is not oral surgery." But whether it is or not, every one is well aware that one department can not do without the other, and the more we can learn of all the sciences the better professional men and women shall we become. The position of dentistry as a specialty of general surgery is justified to a greater degree than in the case of any other specialty, "Surgery," as defined by one of our surgeons, "is that branch of the healing art which takes charge of all those diseases and injuries which require in their treatment the use of instruments and mechanical appliances, operations or especial manual dexterity." To no other specialty does this definition so aptly apply as to our own, and with reference to general surgery as a specialty of medical science, we know that a specialty should be the outgrowth of a liberal professional education—in short, that a specialist should be a man who "knows something of everything and everything of something."

The standard of a profession is raised or lowered by the character and work of its representatives. It rests entirely with them whether they will assert and maintain their claims to their true position, or allow the public to estimate them as it pleases. Better times, however, are coming, better men coming to the front, better thinkers and workers are banding themselves together, resolved to show that dentistry is not beneath, even if it is not above, the medical profession, but that it is a large and distinct branch of medical science requiring not only the same studies but its own special ones as well, and must be recognized and honored as such.

Medical men know too little dentistry, and the dentist too little of general medicine, to be able to examine thoroughly these diseases of the jaws and to treat them as is necessary, seeing that they depend for cure on the attention given by both professions. We, of all people, should know thoroughly the healthy normal appearance of the oral cavity, all it contains, and should therefore be immediately able to detect any pathologic condition. Cultivate thoroughness as early as possible; make a methodical examination, beginning at one tooth, say the left superior third molar, and continue around the arch, and then do the same with the inferior maxilla. Note the condition of the mucous membrane, which shows much of the condition of the roots of the teeth; the tongue, which indicates the state of the alimentary canal; the secretions of the mouth, which aid us in telling the nature of the calcific deposits upon the teeth, and whether the sensitiveness is due to acidity; and then note the color of the lips, and finally the general condition of the patients themselves. If any abnormality be present, examine for the cause with every care, then decide on the treatment and remedy, noting at the same time the idiosyncrasy of the patient. Many of these affections dentists are called upon to treat, and others, after they are diagnosed, we should hand over to the care of the physician. With these cases, whether simple or serious, we should be well acquainted, as we are more likely to see them in their early stages, and by immediate interference we may warn our patient and thus induce him to obtain medical or surgical attention, and thereby save him from long and serious, yes, and often fatal illness.

We are all aware of the difficulty in diagnosing oral tumors. For example, in epithelioma it is far better to urge early surgical interference than to wait until extension by the lymphatics places the patient beyond help. Hence make an early diagnosis; examine every sore, ulcer and spot in the mouth, using every precaution in antisepsis for the benefit of the patient and yourself.

For the reason that the diseases of the jaws are not often discussed, I have ventured to write a few notes on them, to try to show that they bear the same relation to dentistry as do the diseases of the gums. It is a matter of some difficulty to decide how to classify the subject. Perhaps the easiest way will be to discuss first the periosteum and its diseases, and then the close relation it bears to the bone itself will explain how the latter becomes affected.

The alveolo-dental membrane intervenes between the tooth and the bony socket or alveolus. It was formerly supposed to consist of two layers, but the idea has been abandoned since it has been demonstrated that fibers could be traced continuously from the bone to the cementum. The membrane, periosteum or ligament, as it is variously called, consists of connective tissue, between the bundles of which run groups of vessels and nerves, but it contains only a few yellow elastic fibers and therefore can not be stretched much. It is, however, necessary that the tooth should be capable of some degree of movement in its alveolus, for every temporary inflammation by increasing, for the time being, the blood supply of the part, causes the partial extrusion of the tooth, which is so well known to patients as being "longer than the rest." How, then, if we have so few elastic fibers, is movement of the tooth, without tearing the unyielding membrane, rendered possible, unless it be by a special disposition of the fibers? The bony attachment of each band of fibers is nearer the neck than the cemental portion of the tooth, and may be looked upon as suspending the tooth in the alveolus by fasciculi of the periosteum, rather than as resting upon the membrane, and therefore some play is allowed, as the tooth can be raised or lowered until the cemental fibers are visible or lost to view beneath the gum. The fibers are continuous, but there is a slight difference between the portion nearest the bone and that nearest the cementum. At the former place the fibers are larger, while at the latter they become a fine net-work and more cellular. Again, age affects the tissue, for specimens show it to be thinner, especially the cemental portion, but it is not entirely obliterated. The periosteal blood supply is very complex, being derived from the *pulp-artery branching off just before it enters the foramen, and the terminal filaments anastomosing with the arteries that supply the gums. Just at the rim of the alveolus there is a rich plexus formed by union with the arteries of the periosteum and gums, the "gingival plexus," and near the cement there is a capillary plexus. "The blood vessels are most numerous midway between the bone and cementum, or rather nearer the latter." (Tomes.) Thus we may conclude that the teeth have three sources of nourishment, viz.: through the pulp, and through the peridental membrane in its two sources.

The exact nature of the membrane is still far from being satisfactorily explained. Malassez (*Journal British Dental Association*, 1885, p. 484) says "it is a ligament, for these reasons: *a*, if true periosteum existed in such a situation, mastication would be a very painful process, owing to the abundance of nerves; *b*, microscopically the tissue is more ligamentous than periosteal in structure; *c*, mastication produces traction, not pressure; *d*, the attachment to the bone and cementum is precisely like a tendi-

*"The Blood Supply of Teeth," Dr. Hunt, in Dental Review.

nous insertion ; *e*, the distribution of the vessels and nerves in the interstices between bundles of fibers suggests ligament and not periosteum." Ranvier and Kolliker take a similar stand regarding its structure. And yet we know it is true that the membrane is directly continuous with the periosteum covering the rest of the maxilla; that the shock of mastication can be borne on periosteum, as, for instance, in edentulous ridges of the jaw-bone; and again it is certainly not quite a typical ligament, but a typical periosteum, with a specially modified function.

The alveolar walls develop at the same time as the root. Proceeding from the base of the follicle is a line of cells, the formative point of cementum, and another line, the formative organ of the alveolar walls; one develops down to form the socket, the other forms the cementum, the two layers being continuous. Whether the membrane is single or double is a question. Analogy from anatomy, etc., would make us think the true membrane would be in two layers. Demonstration proves it, for on extraction, the root of a tooth is covered by a membrane and the alveolus is lined with a membrane. The alveolar lining is from the bone, and so it must be referred to the osseous system. The formative organ of the cement, can it be the same as that which forms the alveolar walls? Some say it can not, and yet the cementum is like true bone except for the perfection of the Haversian canal, and the lacunæ are irregular in shape, size and distribution. The canaliculi are more numerous from the periosteal side of the lacunæ, fewer run inward toward the dentinal surface, and they contain the remains of the formative "osteoblasts" as we find in bone. The cementum contains "fibers of Sharpey," developed from the formative periosteum, which are calcified bundles of fibrous tissue like those in bone, and therefore why can we not call the cementum a modified bone? Tomes says the cemental organ and that which forms the alveolar walls are not the same; they are closely united but distinct and separable, the external formed from connective tissue with blood vessels, the inner a layer of cells. Dr. Ingersoll says he saw some of Dr. Black's specimens, and one of them was perfect in showing the histologic difference of the two layers. None of the straight lines of the peridental membrane were found in the alveolar membrane. If the membrane is double, two sources of vascular and mineral supply would be expected, from the pulp and from the sub-mucous tissues. Pathology aids us by revealing the difference in exostosis and hypercementosis. Irritation follows the blood vessels, and under the influence of irritants the root may take on an abnormal action; this is one form of exostosis, which latter term should be properly named either ex- or hypercementosis. If the nerves and blood-vessels follow the course of the membrane, and it is not double, why are not both layers affected? Why do we not have both true exostosis or enlargement of the socket and also hypercementosis or enlargement of the roots, consequently tooth-ankylosis? When two roots are exostosed they grow together by impingement, but never the alveolus and root, hence there is no osseous union. Yet again, how often do we find periodontitis go on to periostitis, one being usually primary to the other? Would this not imply they were connected, if inflammation can so readily pass on from one to another? The conclusions I have arrived at by reading and specimens are:

1. The periosteum and the peridental membrane are identical. I prefer the term, periosteum.

2. It is divided into two layers, internal and external, or osteo-genetic and fibrous; the internal, or osteo-genetic, forms the alveolar layer, and the external, or fibrous, forms the cemental layer.

3. The alveolar and cemental membranes are not identical.

4. The two layers have different functions; one forming and lining the alveolus, the other surrounding and holding the root.

5. The cement appears to be developed from the periosteum, and not from the dentine or odontoblast cells, as under the microscope it has the same appearance as bone, except that the lacunæ and canaliculi are not so perfectly developed.

When cementum is required to be very thick, as in herbivorous animals, where it is destined to cover the whole tooth, Magitot says, it first becomes the seat of a formation of hyaline cartilage, which ossifies like any other bone developed after the intracartilaginous method. Where it is to be thin and only cover the crown, as Nasmyth's membrane, it is the seat of ordinary intramembranous ossification.

6. Pathology shows the same fact; there is no union between the root and the alveolus.

7. The rapid mineral absorption of the root without exfoliation is due to the special nature of the cemental membrane as distinct from the membrane of the socket, and has been microscopically proved.

8. It is a great deal better to consider the peridental membrane as periosteum divided into two layers.

| | | |
|---|---|---|
| Periosteum | External (fibrous) layer, continued as the cemental layer.......................... | peridental. |
| | Internal (osteo-genetic) layer, continued as the alveolar layer .......... ..... | membrane. |

The structure: white, fibrous tissue, cells, blood vessels, nerves, little elastic tissue and no fat. Thicker at the neck than at the root. Fibers of periosteum run downward and inward to the root, so the more pressure on the tooth the more tense the fibers. Structurally, there is one common periosteum for tooth and alveolus, the fibers at the cemental surface being looser than near the bone. The fibers attached to the bone are arranged into bundles; thus some parts are seen to be denser than others. In the reticular or cemental fibers, cells or osteoblasts are seen and also at the bone surface. Corpuscles are also dotted all throughout the periosteum. The fibers of the periosteum at the neck blend with the fibrous tissue of the gum, and with the periosteum of the alveolar border. Pulpitis leads to periodontitis or periostitis by free communication from vessels of the gum.

[*To be Continued.*]

